# Original Translations

**Published:** 1884-01

**Authors:** 


### Original Translations.

By A. Lagorio, M.D.

### Extraction of a Large Foreign Body, Situated in the Rectum.

Dr. Josè Senilla Victorio reports an operation of this kind (*Revista Medico-quirurgica*) performed in 1882, by Dr. Mantes de Oca, in the Hospital of Buenos-Ayres, on a man named E. Chaillot, aged 35 years, and of good constitution. This individual was said to have once placed a medicated bougie in the rectum, and to have then felt a special sensation accompanied by an erection of the penis and an ejaculation; this sensation was more pronounced when he touched the anterior surface of the rectum. Having discovered this means of obtaining a systematic evacuation, he frequently resorted to the bougie, increasing it in volume in order to obtain the desired effect. Little by little he used instruments of greater size and of a different composition, until he employed finally a porcelain insulator, such as are generally seen on telegraph posts; they have a smooth surface and a groove near the superior extremity; at the base they have a deep concavity. The dimensions of these bodies are: height, 10 cent.; superior diameter, 5 cent.; inferior diameter, 8 cent.; circumference at the base, 29 cent. The insulator did not have a length sufficient to insure its being held during its introduction, and when introduced it became impossible to withdraw it, although he had placed a twine around in the groove. Different attempts at extraction were made, with no success. A small fragment was broken off around its base. The forceps, the Liston pincette, were applied, but they only served to break off fragments. The condition of the patient was very bad. A capital operation was decided upon. The patient was chloroformed, the operator introduced his oiled hand in the rectum and successively used the Pajot's forceps, a curved trocar, and a linear constrictor of Chassaignac. Nine attempts were then made in three hours, but the body remained in the intestine; gangrene had already set in; rest was ordered and opium given to ease the pains, but the operation became urgent and Montes de Oca returned after eight hours. Chaillot was again placed under the influence of the anæsthetic. The surgeon then opened the abdominal cavity and incised the large intestine at the inferior extremity of the sigmoid flexure, and extracted the body. The borders of the incised colon were sutured, the abdominal cavity cleansed and closed. The operation lasted three hours; syncope occurred and was overcome by artificial respiration. Some hours after the patient died. At the autopsy the injured parts presented a very satisfactory condition, the sutures were perfect. The death could not be attributed to the operation itself, but to the gangrene already commenced before the operation, to the shock, and also to the repeated use, at short intervals, of an anæsthetic agent causing a true intoxication.

### QUEBRACHO AND ITS ALKALOIDS.

Huchard and Eloy read a paper before the Therapeutical Society of Paris on the physiological, toxic, and therapeutical properties of quebracho and its alkaloids. *Quebracho Blanco* is a beautiful tree, growing in the Argentine Republic. The bark, which is the only active part, is there used as a tonic, febrifuge, and anti-asthmatic. Penzoldi experimented with it on animals, and found that in toxic doses it produced death, with paralysis of the limbs, intense dyspnœa, and asphyxic convulsions. Quebracho possesses six alkaloids: aspidospermine, asperdospermative, aspidosamine, quebrachine, hypoquebrachine, and quebrachamine. Besides these, also, quebrachol, of an alcoholic character. The aspidospermine of commerce, used by all experimenters, is a mixture of all the alkaloids. The authors have found that all these different alkaloids have different properties, and almost antagonistic. They have employed the alkaloids prepared by Tauret, and experimented with them in the laboratory of Brown-Sequard, on dogs and rabbits. Aspidospermine increases the amplitude of the respiratory movement, and accelerates this movement in all the animals. These modifications of respiration come on five to ten minutes after the subcutaneous injection of 4 to 5 centigr. of the chlorhydrate of aspidospermine. In toxic doses, the respiration becomes embarassed, and the animal dies in convulsions. Aspidospermine does not kill by asphyxia. In the autopsy of all animals the blood was found to be vermillion red, as in poisoning by prussic acid. The temperature is lowered from 39° to 36° 5 C.

Quebrachine has an action entirely different from that ot aspidospermine. It seems that it acts as a paralyzer of the respiratory muscles. Greatly more toxic than aspidospermine, it produces death by asphyxia, with a black coloring ef the blood and elevation of temperature. An animal died in five minutes after an injection of three drops of a 4 per cent. solution of quebrachine, hypoquebrachine, and aspidospermine. Huchard concluded that aspidospermine is a modifier of the respiratory movements, an agent capable of arresting changes, and a refrigerant. These different properties are important in the diseases which produce dyspnœa, apnœa, and asphyxia.

Constantine Paul made an observation that quebracho contains much tannin; he had used it as a topical remedy in cases of vaginitis, blennorrhagia, metritis, etc.—(*Le Praticien.*)

MYXOSARCOMA OF THE PLEURA.

Dr. Hofmokl (*Soc. des Médic. de Vienne*, 1883) presented an observation of a rare affection of the pleura. The subject was a child three and a half years old. Had diphtheria when six months old, and a bronchitis when two and a half years. Last February contracted again a bronchitis, and recovered without presenting any serious thoracic symptoms. On the 12th of March, 1883, the author noticed some symptoms of uneasiness, a marked paleness, and an acceleration of the respiratory movements. On examining the chest he found at the base of the left side and posteriorly a marked flatness, with a diminution of the vesicular murmur, diminution of the vibrations and a relative immobilization of the thoracic cavity. The heart is not displaced; pulse frequent; temperature 38.5 C. The doctor readly diagnosticated a pleural effusion of the left side.

Under the influence of quinine the temperature was lowered, the general condition seemed to improve, and the little patient regained his sleep and appetite. After eight days the fever returned; temperature 39° C. The flatness increased, with a thoracic bulging anteriorly and to the left, and the heart is displaced. On the 14th of April, on account of the progressive dilatation of the chest and the accumulation of the signs of the supposed effusion, the author performed thoracentesis. He only obtained a little sanguineous fluid. After the operation the dyspnœa increased. On the 17th he performed a second puncture with the same effect. Sharp pains supervened, with an increase of the dyspnœa, the doctor then decided to perform resection of a rib. The patient was anæthetized and a fragment of the right rib (1½ centimeters) was resected. The operation gave the same result as the former. The author then introduced a finger through the openings, and detected a tumor surrounded by softened sanguineous clots in great quantity. Notwithstanding the Lister dressing the patient succumbed after three days. At the autopsy it was learned that the neoplasm had its point of origin in the left pleura, and the microscope demonstrated the elements of a myxosarcoma.—*Archives Générales de Médecine.*

THE ÆTIOLOGY OF CONGENITAL DEFORMITIES OF THE BULBUS OF THE EYE.

Among women an "impression" in pregnancy is generally believed, but physicians have no reason to do so. The attention of scientists being directed to the development of eye fissure and the consequent anomalies of the eyebulb, it was found that the formation of the coloboma is an intra-uterine inflammatory process of the fœtus, which originates in the retinal fissure at a time of development when this fissure begins to close and which attacks the choroidea and afterwards the retina. Thalberg at St. Petersburg, reports eighteen cases of congenital deformity among 2,678 children. There was eight times anophthalmus, four times mikrophthalmus, and six times coloboma of the iris and choroidea. In the autopsy of two children he examined the eyes and found that there had been been an intra-uterine inflammatory process in the eye fissure, which caused an interruption in the development of the choroidea and iris, and thus produced coloboma. The most important pathological changes in utero involved especially the retina on the entrance of the optic nerve, which were produced by exuberant growths of cells. The inflammatory process had started from this point and it had originated at a time when the eye fissure was not yet closed. Around the optic nerve and inside of the coloboma the inflammatory process had caused a thickening and degeneration of the entire membrane with destruction of the nerve, and ganglion, cellular stratum and of the cells of the pigmented epithelium of the retina. There was furthermore by nutritive interruption a degeneration and partial resorption of the basal filaments of the posterior pole of the lens, but the retina was preserved in the coloboma. The differentiation between the layers is begun as generally known, before the closure of the fœtal retinal fissure. But in this case the inflammation had started before the closure and continued at the closure, producing a folding of the retina and a conglomeration of the accumulating cells of the pigment epithelium. The development of the choroidea was but rudimentary, there being round cells in the place of the choroidea or external layers of non-pigmented spindle cells. It is most probable that this inflammatory process produces sometimes anophthalmus, and the process is evidently a local one, the relation to the maternal parts being not established. In one case, a woman in the fifth month of pregnancy dropped angelica tincture into the right eye instead of a solution of zinc which caused considerable pain and conjunctivitis. The child had mikrophthalmus and coloboma of the iris of the left eye and the microscopical examination showed a closure of the retinal fissure in the seventh week.—*St. Petersburg Medical Wochenscrift.*

### DIFFERENTIAL DIAGNOSIS OF LUPUS, LEPRA, AND CANCER IN THE THROAT.

The Spanish physician, Ramon de la Sotay Lastra, gives the following essential points on these diseases: Only a very experienced eye is able to distinguish the frequently occurring forms of lepra, lupus and cancer in the throat. All three have the peculiar form of nodules; all three are liable to become ulcerated. In two, will follow cicatrization, while but in one, there is a probability of a cure.

*Lepra* is never localized in the throat, and there is always first a disease of the skin. The first appearance in the throat is marked by dark red spots on the mucous membrane of the palate, pharynx and uvula. This redness changes soon to a whitish color, and but a few blood-vessels are visible. The eruptive state is characterized by opaque white nodules, which are soft and at first sensitive, but later removable without pain. On the tongue they appear as papillar ulcerations, on the palate and pharynx in the forms of chains; the uvula having then a rough surface; the epiglottis, ary-epiglottis and the ventricles seem to be encircled by nodules; the inner pharynx has a granulated surface, and the glottis seems to be closed. All the diseased parts swell considerably, the epiglottis looking like an œdematous preputium. The breath is fetid. Finally, after certain intervals, there are formed small confluent ulcers which cover a large part of the tongue, the pharynx, and larynx. They are of a round form, and resemble syphilitic plaques; others are marginated and discolored, the ground being finely granulated. The first appearance of the ulcers is in the palate; then the uvula is attacked and entirely destroyed; the epiglottis, its folds and ary-cartilages are likewise destroyed, and the vocal cords are swollen and hardly movable. At this period there exists a close stenosis, the voice is altered, the breath fetid, and pieces of the larynx are expectorated.

*Lupus* is liable to appear in the throat without formation of nodules in the skin, but this is seldom. The nodes in the throat are seen as elevated spots without discoloration, and they are of a hard elastic consistence. They grow slowly, and the voice and deglutition are gradually altered, but it takes a long time before granulations are seen. The latter resemble large fissures, and they have red elevated margins. If there is complete formation of scars, the latter are irregular, in some places elevated, in others depressed, and they contract without deformation of the parts.

*Primary Cancer* of the throat appears often in old age, in apparently healthy persons. It is marked by congestion and swelling, and there is hardly any pain. But soon there appear nodules, of a grey or red color and of variable size, but which grow slowly and are accompanied with intense pains in the ears and eyes. These nodules change quicker than in lupus to granlating surfaces, covered with thick mucus, which is mixed with blood and pus. There is considerable œdema in the adjacent parts and an abundant secretion of saliva. Here the progress is fatal.—*Revista especial de Sifiliografia y Dermatologia.*

TREATMENT OF DIPHTHERIA WITH THE CYANIDE OF MERCURY.

Dr. Greuholm recommends, among the new remedies for diphtheria, the following solution :

℞—Cyanide of Mercury, - - - 10 centigr.
Peppermint Water, - - - 100 grams.

M. Sig. A teaspoonful every hour.

Dr. Seldéin has used this formula; but was obliged to use a weaker solution on account of nausea, of stomatitis, and of great disgust:

℞—Cyanide of Mercury, . . . 1 centigr.
Water, . . . . . . . . 100 grams.

Add a little honey to correct the taste.

With this remedy he has cured 34 patients out of 37. The patients, if old enough, will gargle the above solution. At the same time is given ice, milk, stimulants (as Hungarian wine), and turpentine.—(*Revue Médicale.*)

THERAPEUTIC ATAXIA OF HYSTERIA.

Huchard has found that certain remedies, such as the iodide of potassium and the salicylate of sodium, are eliminated very slowly in hysteria. He considers this sort of theurapeutic ataxia of hysterics due to three principal causes: To the mental state, to the atony of the digestive organs and to the functional troubles of the kidneys.—*Le Praticien.*

